# Identification of KIF4A as a prognostic biomarker for esophageal squamous cell carcinoma

**DOI:** 10.18632/aging.203585

**Published:** 2021-11-14

**Authors:** Lingwei Wang, Gang Liu, Enkhbat Bolor-Erdene, Qinchuan Li, Yunqing Mei, Lei Zhou

**Affiliations:** 1 Affiliated Zhongshan Hospital of Dalian University, Dalian 116001, China; 2 East Hospital of Tongji University School of Medicine, Shanghai 200120, China; 3 Department of Cardiothoracic Surgery, Xiangyang No. 1 People’s Hospital, Hubei University of Medicine, Xiangyang 441000, China; 4 Department of Cardiothoracic Surgery, Tongji Hospital, School of Medicine, Tongji University, Shanghai 200065, China

**Keywords:** ESCC, KIF4A, proliferation, migration, invasion

## Abstract

Esophageal squamous cell carcinoma (ESCC) is the most common and aggressive tumor worldwide, and the long-term survival of these patients remains poor. Three databases (GSE17351, GSE20347, and GSE100942) were obtained from Gene Expression Omnibus, and 193 differentially expressed genes including 56 upregulated and 137 downregulated genes were identified by paired test using limma R package. Then, functional enrichments by gene ontology and Kyoto Encyclopedia of Genes and Genomes analyses showed these genes were mainly related protein digestion and absorption, and IL-17 signaling pathway. We then constructed a protein-protein interaction network and cytoHubba module to determine the six hub genes and overall survival analysis of the six hub genes were evaluated by UALCAN and GEPIA2 analysis. Ultimately, the experimental results confirmed the KIF4A was overexpressed in the ESCC tissues and cell lines compared with the normal esophageal mucosal tissues and was linked to poor prognosis. Moreover, we also revealed that KIF4A facilitates proliferation, cell cycle, migration, and invasion of ESCC *in vivo* and *in vitro*. Overall, these findings demonstrated that KIF4A could serve as diagnostic and prognostic biomarkers and may help facilitate therapeutic targets in ESCC patients.

## INTRODUCTION

Esophageal cancer (EC) is an aggressive malignant tumor globally, which ranks seventh in morbidity and fourth in mortality among all tumors. Meanwhile, obvious geographical differences are found in esophageal cancer and most of which are in East Asia [1, 2]. Among all the histological types of esophageal carcinoma, esophageal squamous cell carcinoma (ESCC) is the most frequent worldwide [3]. Recently, although various therapies such as esophagectomy, chemotherapy, radiotherapy, and immunotherapy are frequently used to treat ESCC, the long-term outcome remains poor, with a five-year survival rate in the range of 15–25% [4]. ESCC is a highly heterogeneous disease involving changes in multiple gene expression patterns. Thus, revealing ESCC initiation mechanisms and potential prognostic molecular markers are essentially required to improve patients’ outcomes with ESCC [5].

The kinesin superfamily (KIFs) is a major component of the intracellular transport system, responsible for cell morphology and physiological function realization [6]. Kinesins also play essential roles in cell division, cell motility, spindle assembly, chromosome aggregation, and separation [7, 8]. KIFs proteins are classified into 14 subfamilies [9]. Of these, kinesin superfamily member 4A (KIF4A) is an essential chromosome-associated molecular motor, containing an N-terminal motor, coiled-coil, and C-terminal loading regions. It holds crucial functions in DNA repair and replication [10]. KIF4A maps to Xq13.1, encodes a protein comprising 1232 amino acids, and mainly localized in the nucleus. KIF4A plays a pivotal role in DNA repair and DNA replication and is also essential for regulating chromosome segregation and mitotic spindle organization during mitosis [11]. Importantly, genetic stability is affected by aneuploidy, which is strongly correlated with tumorigenesis [12]. Interestingly, high KIF4A expression was observed in certain cancer types, such as lung cancer [13], oral cancer [14], breast cancer [15], and hepatocellular carcinoma [16]. In contrast, low expression was observed in gastric cancer [17] and osteosarcoma [18], which was observed to inhibit tumor cell proliferation, cell cycle, migration, and invasion. Nevertheless, the underlying molecular mechanisms of KIF4A in ESCC remain unknown.

In recent decades, microarray assays and bioinformatic analysis have been extensively used to study the molecular landscapes of tumors at multiple levels, such as somatic mutations, copy number alterations, and epigenetic alterations [19]. In our study, three datasets were downloaded from the Gene Expression Omnibus database, and they contain 26 pairs of tissues. The sva and limma R packages were utilized to remove the batch effects across different platforms and integrate them to obtain differentially expressed genes (DEGs) between paired samples. The gene ontology (GO) functional annotation and Kyoto encyclopedia of genes and genomes (KEGG) pathway analysis were then performed. Protein-protein interaction (PPI) network and module analyses were performed by Cytoscape software. Meanwhile, pathological staging and OS analysis of the six hub genes were evaluated by UALCAN and GEPIA2 analysis. Finally, we identified that KIF4A might be a potential biomarker for ESCC. To further determine the specific mechanism of KIF4A in ESCC, we found that the expression of KIF4A in ESCC tissues and cell lines was higher, and its high expression level predicted worse survival in ESCC patients. Besides, we demonstrated that KIF4A accelerated ESCC proliferation *in vitro* and *in vivo*. Furthermore, KIF4A promoted metastasis by attenuating epithelial-mesenchymal transition (EMT) in ESCC cells. In short, we propose for the first time that KIF4A overexpression could serve as a novel prognostic biomarker and potential therapeutic target toward ESCC therapy.

## RESULTS

### Microarray data

GSE17351, GSE20347, and GSE100942 databases were downloaded and analyzed. If a dataset needs to be normalized, this can be accomplished using the normalize Between Arrays function in the limma R package, as shown in Supplementary Figure 1. In this regard, GSE100942 and GSE17351 did not need to be normalized, and GSE20347 needed to be normalized.

### Differentially expressed genes between paired samples of primary tumors and normal tissue by paired test

**Figure 1:**
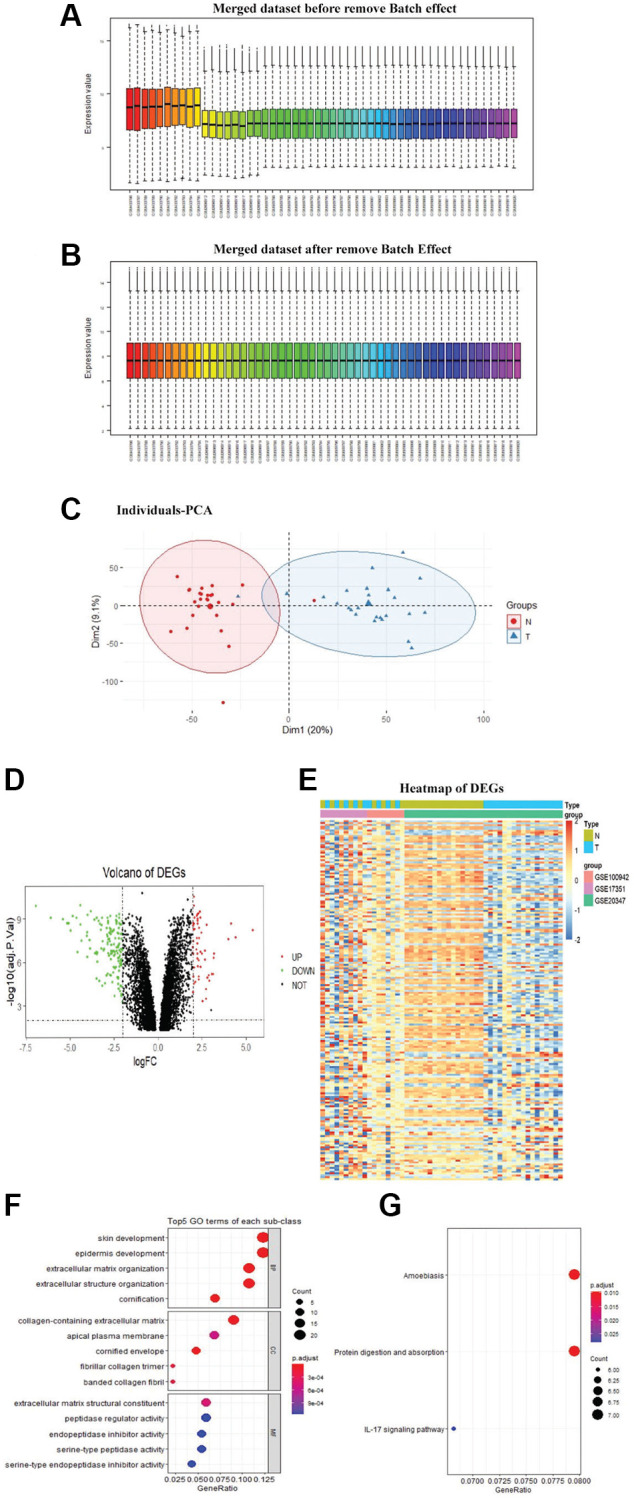
**Identification of DEGs.** The merged dataset before removed the batch effect (**A**). The merged dataset after removed the batch effect (**B**). PCA was done for the merged dataset for dimensionality reduction and quality control (**C**). Volcano plot of DEGs, the red dots represent the upregulated genes and the green dots represent the downregulated genes based on an adjusted *P*-value < 0.001 and | logFC | ≥ 2, the black spots represent genes with no significant difference in expression (**D**). Heatmap of DEGs, red representing high relative expression, and blue representing low relative expression (**E**). DEGs with the top 5 enriched GO terms (**F**) of each subclass and KEGG (**G**) pathway enrichment analyses of DEGs (*P* < 0.05)

The sva and limma R packages were utilized to remove batch effects (Figure 1A, 1B) across the different platforms and integrate datasets to obtain DEGs between paired samples by paired test. Principal component analysis (PCA) was done for the merged dataset for dimensionality reduction and quality control (Figure 1C). A total of 193 DEGs, including 56 upregulated and 137 downregulated genes, were identified. The Volcano map and heatmap of DEGs were depicted in Figure 1D–1E.

### GO Functional and KEGG Pathway Enrichment Analysis of DEGs.

GO analysis showed that DEGs were mainly involved in skin development, collagen-containing extracellular matrix, and extracellular matrix structural constituent (Figure 1F). The enriched pathways obtained in KEGG analysis were amoebiasis, protein digestion and absorption, and IL-17 signaling pathway (Figure 1G).

### PPI network construction and hub gene selection

**Figure 2:**
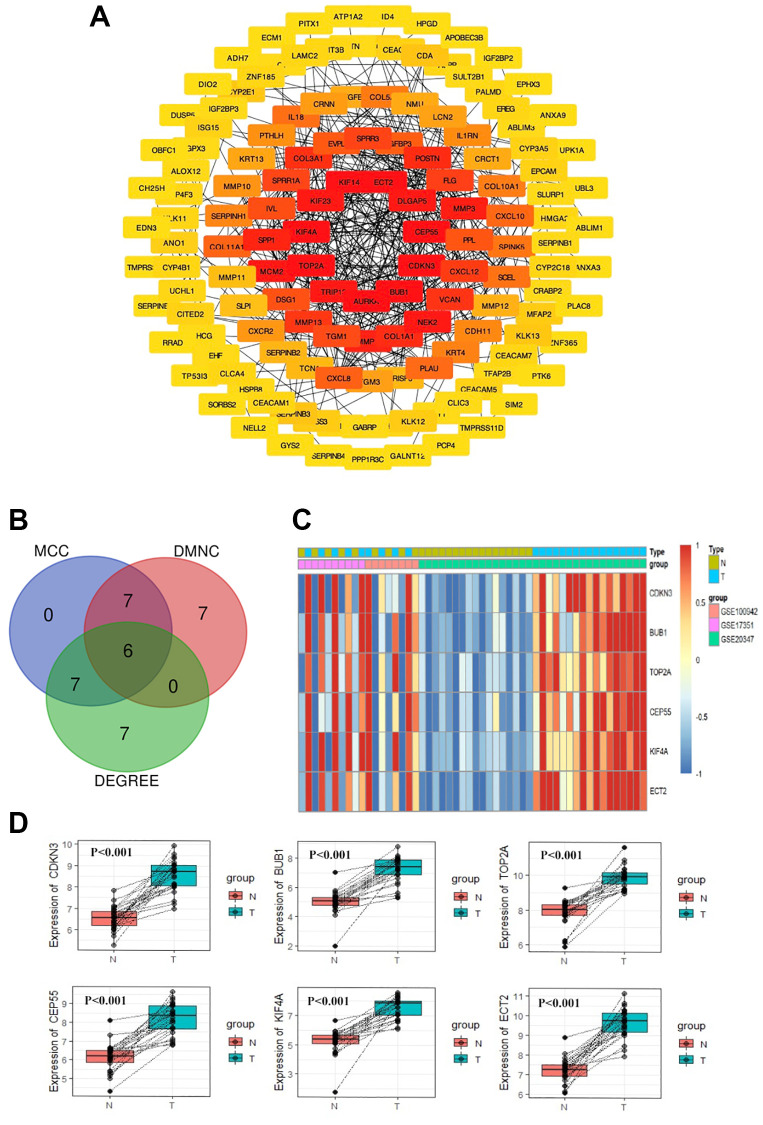
**PPI network construction and Hub gene selection.** The PPI network of DEGs was constructed using Cytoscape (**A**). The Venn diagram of the overlapped 6 hub genes from the topological algorithms in the cytoHubba module (**B**). The heatmap of 6 hub genes (**C**). The expression difference of the 6 hub genes between tumor tissues and normal tissues by paired test from GEO (**D**).

As displayed in Figure 2A, the most significant module and PPI network of DEGs was obtained by STRING and Cytoscape. Hub gene screening was accomplished using cytoHubba module. We then applied the three most common topological algorithms (Maximal Clique Centrality, Density of Maximum Neighborhood Component and Degree) to filter the top 20 genes, and finally, we obtained six hub genes. Figure 2B and Figure 2C displayed the Venn diagram and heatmap of six hub genes, respectively. The expression difference between paired samples of tumor and normal group of the six genes from GEO database is demonstrated in Figure 2D. The six hub genes, namely CDKN3, BUB1, TOP2A, CEP55, KIF4A, and ECT2, were all upregulated in ESCC. The names, abbreviations, and functions for these hub genes were shown in Table 1.

**Table 1:** Functional roles of 6 hub genes.

**No.**	**Gene Symbol**	**Function**
1	CEP55	Mitotic exit and cytokinesis
2	TOP2A	Controlling of topological states of DNA by transient breakage and subsequent rejoining of DNA strands
3	KIF4A	Chromosome segregation during mitosis,the formation of an organized central spindle midzone and midbody and for successful cytokinesis
4	BUB1	It is essential for spindle-assembly checkpoint signaling and for correct chromosome alignment. Acting as a substrate for anaphase-promoting complex or cyclosome (APC/C) in complex with its activator CDH1 (APC/C-Cdh1)
5	ECT2	Guanine nucleotide exchange factor (GEF) that catalyzes the exchange of GDP for GTP
6	CDKN3	May play a role in cell cycle regulation. Dual specificity phosphatase active toward substrates containing either phosphotyrosine or phosphoserine residues. Dephosphorylates CDK2 at ‘Thr-160’ in a cyclin-dependent manner.

### Analysis of hub genes as prognostic indicators of survival in ESCC

**Figure 3:**
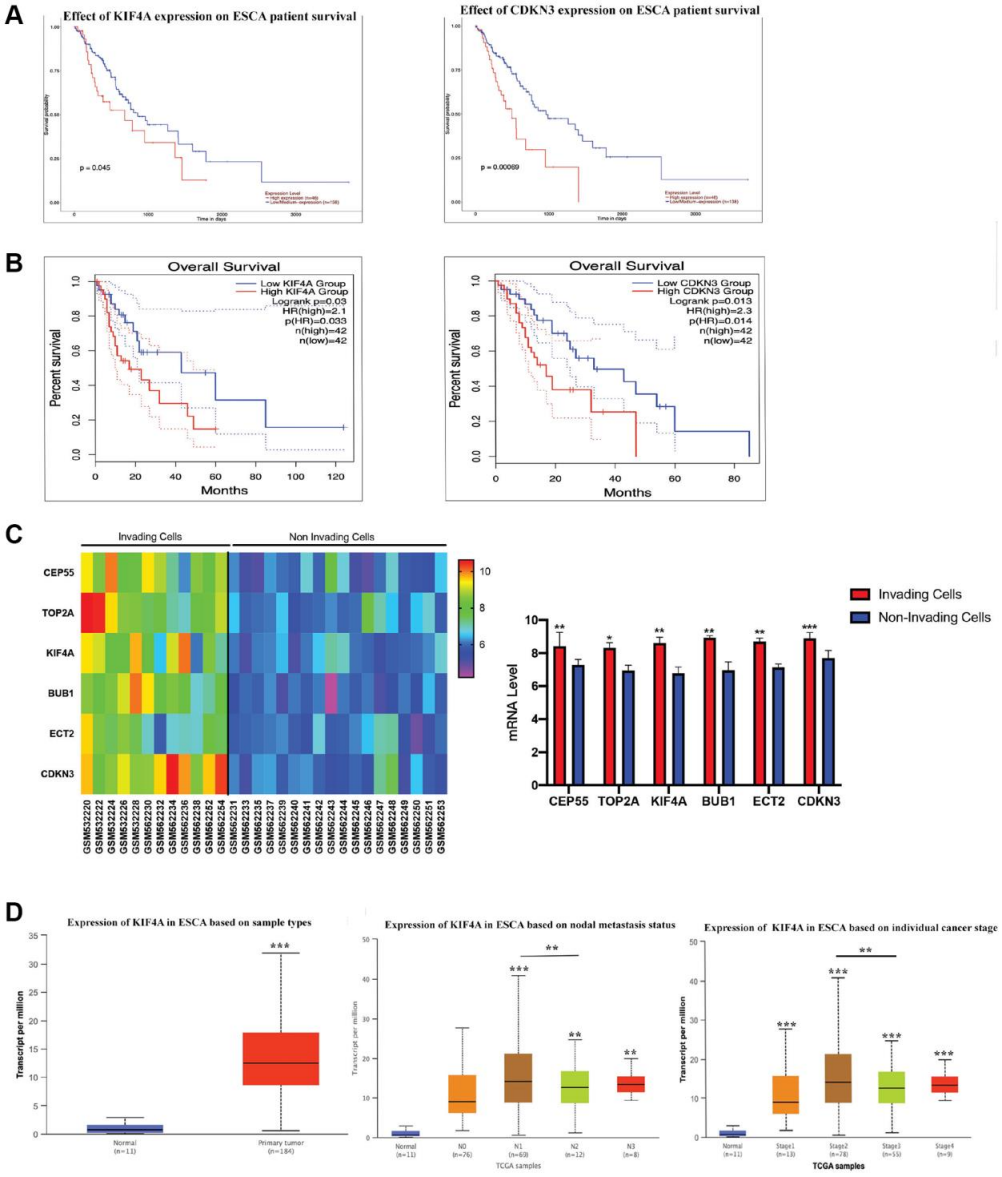
**The survival analyses for hub genes.** CDKN3 and KIF4A were identified to have an adverse effect on the prognosis of overall survival for patients with ESCC by UALCAN (**A**) and GEPIA2 (**B**) analysis. Gene expression of 6 hub genes between invading and non-invading cells based on the GSE21293 (**C**). Association between clinical staging, nodal metastasis status of ESCC and expression levels of the KIF4A by UALCAN analysis (**D**).

To determine whether hub genes were linked to ESCC patients’ survival, six prognostic hub genes were identified and survival analysis was further performed. Only high KIF4A and CDKN3 expression were correlated with poor OS in ESCC patients (Figure 3A–3B, and Supplementary Figure 2). However, CDKN3 demonstrated that it might promote proliferation, migration and invasion of ESCC via the AKT pathway [44]. In order to study whether these six hub genes affected ESCC metastasis and invasion, the expression levels of these genes were analyzed through the GSE21293 dataset which containing invading and non-invading EC cells [17]. Primary esophageal cells were established from surgically resected specimens of ESCC patients (*n* = 35). The present study showed that the expression levels of all six hub genes were up-regulated in invading cells (Figure 3C). In conclusion, we determined that KIF4A was our research target. Meanwhile, for N categories, high KIF4A expression was tightly correlated in patients with N1, N2, and N3 stages. Moreover, KIF4A expression was closely associated with stage I, II, III, and IV (Figure 3D). All in all, among these six hub genes, KIF4A may be a prognostic indicator of ESCC.

### KIF4A was upregulated in ESCC and indicated poor prognosis

**Figure 4:**
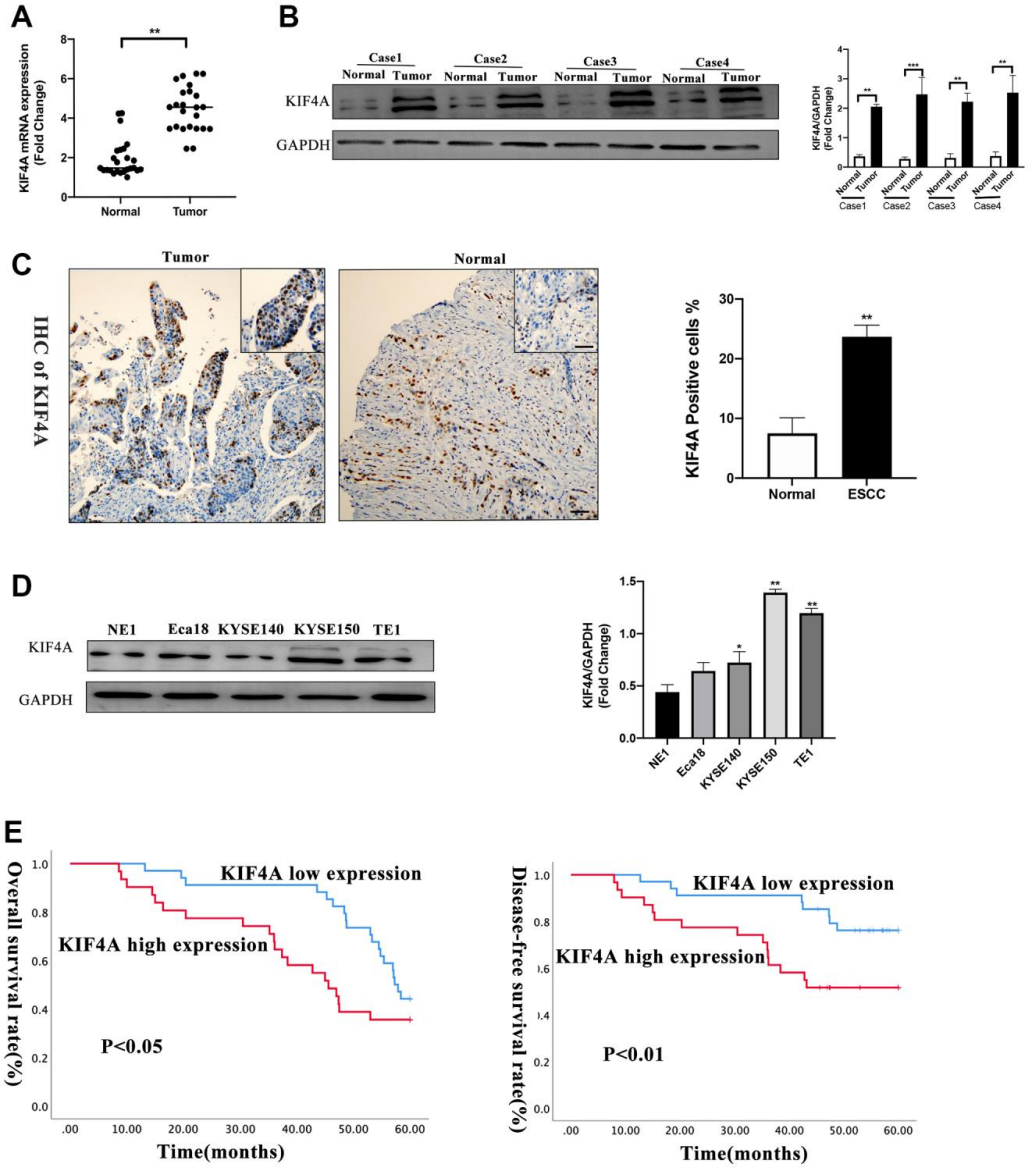
**KIF4A was upregulated in ESCCs and ESCC cell lines.** KIF4A expression was compared by qRT-PCR (**A**), WB (**B**), immunohistochemistry (IHC) (scale bar 50 um) (**C**) between tumor and corresponding non-tumor tissues in 25 ESCCs. Expression of KIF4A was detected in the ESCC cell lines by WB analysis compared with an immortalized esophageal epithelial cell line NE1 (**D**). Kaplan–Meier analysis showed the overall survival rate and disease-free survival rate of ESCC patients stratified by KIF4A expression (**E**). Data were presented as mean ± SEM. ^*^p < 0.05, ^**^p < 0.01, ^***^p < 0.001.

To further explore KIF4A expression between ESCC and paired adjacent normal tissues, quantitative real-time PCR (qRT-PCR) was used to confirm the mRNA levels of KIF4A, and the result showed that in 25 paired of tumor tissues, KIF4A mRNA levels were significantly higher than those in adjacent normal tissues (1.7 vs. 5.3-fold changes) (Figure 4A). Levels of KIF4A proteins were also detected in four primary ESCC tissues and matched adjacent normal tissues by western blotting (WB). The findings revealed that KIF4A protein levels were higher in primary ESCC tissues than in paired adjacent normal tissues (Figure 4B), and immunohistochemistry (IHC) analysis demonstrated similar results (Figure 4C). WB analysis also indicated that KIFA expression was higher in all ESCC cell lines than immortalized esophageal epithelial cell line NE1 (Figure 4D). Based on IHC results, 34 cases were defined as low KIF4A expression, while 31 cases were identified as high expression. The KIF4A protein expression and clinicopathological features are summarized in Table 2. The results indicated that KIF4A protein expression levels were markedly correlated with tumor diameter (*P* = 0.021), degree of infiltration (*P* = 0.012), lymph node metastasis (*P* = 0.047), distant metastasis (*P* = 0.009) and TNM stage (*P* < 0.001). Then, we analyzed 65 human ESCC samples and the results showed that higher KIF4A expression relate to a poor OS (*P* < 0.05), and our samples confirmed that patients with higher KIF4A expression predicted a decreased OS (*P* < 0.05) and DFS (disease-free survival, *P* < 0.01) (Figure 4E). Multivariate analysis exhibited that KIF4A was an independent predictor (*P* = 0.001) (Table 3).

**Table 2:** KIF4A staining and clinicopathological features of 65 ESCC patients.

Variables*		**KIF4A staining**			
		**Low**	**High**	**Total**	***P* value**
Age	≤56 years	15	13	28	0.748
	>56 years	19	18	37	
Gender	Male	16	15	31	0.568
	Female	18	16	34	
Tumor Size	≤5 cm	19	13	32	0.021
	>5 cm	15	18	33	
Depth of invasion	T1–2	18	14	32	0.012
	T3–4	16	17	33	
Lymph node metastasis	N0	26	19	45	0.047
	N1–3	8	12	20	
Distant metastasis	M0	31	26	57	0.009
	M1	3	5	8	
TNM Stage	I	25	17	42	<0.001
	II	5	9	14	
	III	4	4	8	
	IV	0	1	1	

**Table 3:** Multivariate Cox regression analysis on 5-year overall and disease-free survival of 65 ESCC patients.

Variables^*^	**Overall survival**		**Disease-free survival**	
	**HR (95% CI)**	***P***	**HR (95% CI)**	***P***
KIF4A	6.371 (2.376–17.082)	<0.001	4.752 (1.912–11.810)	0.001
Age	0.884 (0.417–10875)	0.748	0.903 (0.421–1.934)	0.792
Gender	1.251 (0.581–2.692)	0.568	1.204 (0.558–2.601)	0.636
Tumor size	6.358 (1.320–30.631)	0.021	5.914 (1.226–28.534)	0.027
Depth of invasion	14.475 (1.805–30.072)	0.012	11.927 (1.523–93.409)	0.018
Lymph node	2.297 (0.860–6.136)	0.097	2.577 (0.968–6.856)	0.058
Distant metastasis	3.397 (0.762–15.130)	0.109	2.884 (0.654–12.731)	0.162
TNM stage	8.932 (3.085–25.860)	<0.001	10.537 (3.433–32.340	<0.001

^*^KIF4A: low vs. high; Age: ≤56 years vs. > 56 years.

### KIF4A facilitated the proliferation of ESCC *in vitro*

**Figure 5:**
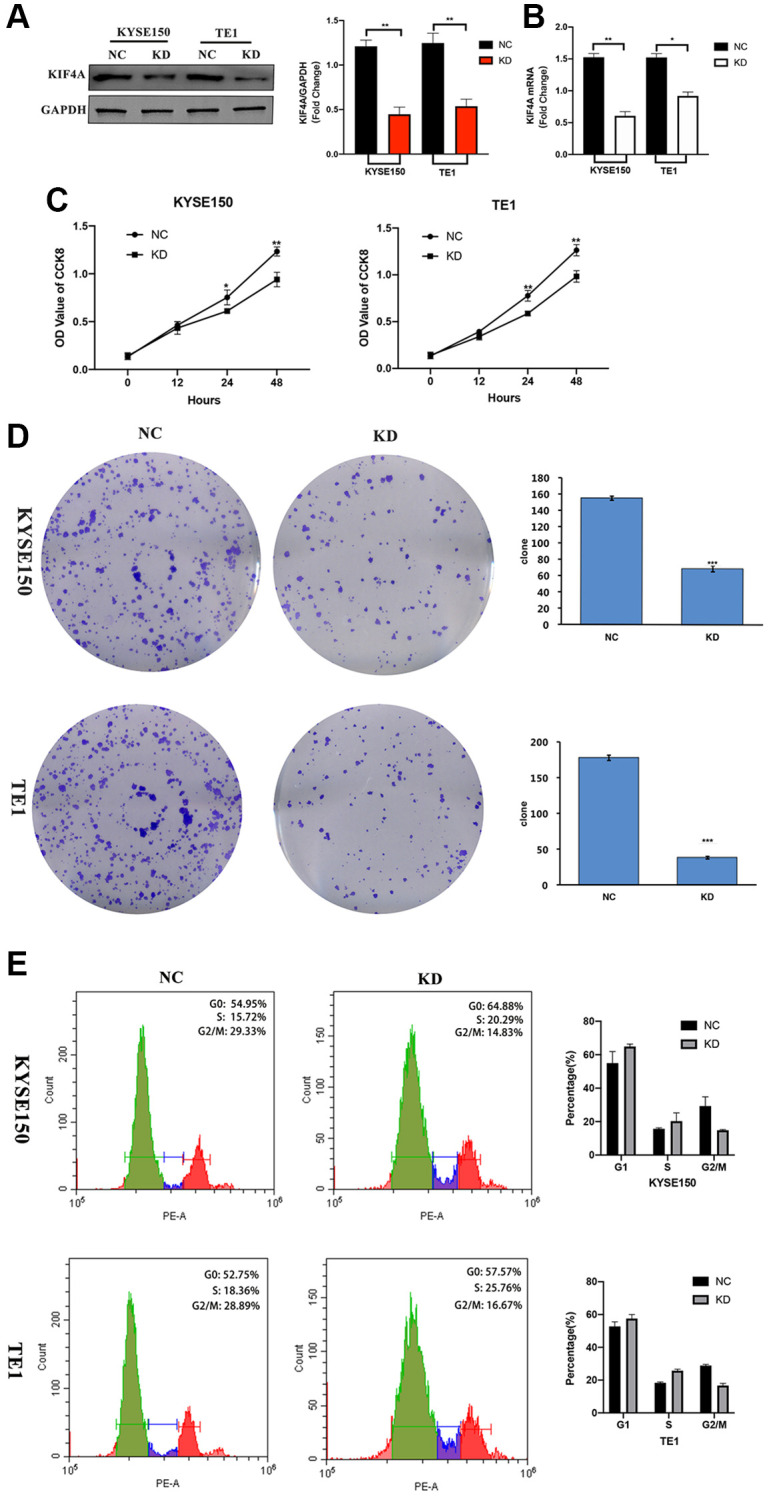
**KIF4A accelerated cell cycle and proliferation of ESCC cells.** KYSE150 and TE1 ESCC cells were transfected with shRNAs specifically targeting KIF4A or scrambled shRNA control (NC). The effective knockdown of KIF4A in these ESCC cells was confirmed by WB (**A**) and RT-qPCR (**B**). Cell proliferation of these KIF4A knockdown and NC cells were detected by CCK8 assay (**C**). Colony formation was detected by single-cell clone assay (scale bar 50 um) (**D**). Representative images of cell cycle distributions of KYSE-150 and TE-1 cells transfected with control or KIF4A shRNAs for 48 h were determined by flow cytometry (**E**). Results are representative of three independent experiments performed in triplicate. The data are presented as the means ± SD. Statistically significant difference: ^*^P < 0.05, ^**^P < 0.01, ^***^P < 0.001, *n* = 3.

In order to study the potential role of KIF4A play in the progress of ESCC, we used lentiviral KIF4A short hairpin RNAs (shRNAs) stable knockdown the expression of KIF4A in KYSE150 and TE1 cells, and a scrambled shRNA was used as a negative control (NC). The expression of KIF4A in cells was evaluated by WB (Figure 5A) and qRT-PCR (Figure 5B). Based on these results, CCK-8 cell assay showed that the proliferation capability of the two cell lines was significantly reduced after KIF4A knockdown (Figure 5C). Colony formation experiment confirmed that KIF4A knockdown cells produce lower numbers and smaller colonies (Figure 5D). In addition, we have further investigated whether exhaustion of KIF4A can lead to cell cycle stoppage. The results showed that KIF4A knockdown can trigger the G2/M phase arrest of KYSE150 and TE1 cells, resulting in a significant increase in the ratio of G2/M phase cells (Figure 5E). In summary, these data suggested that KIF4A might be essential for proper mitotic progression. However, KIF4A had no significant effect on apoptosis in both ESCC cell lines (Supplementary Figure 3). These results suggest that KIF4A promote ESCC cell proliferation.

### KIF4A promote cell migration and invasion of ESCC

**Figure 6:**
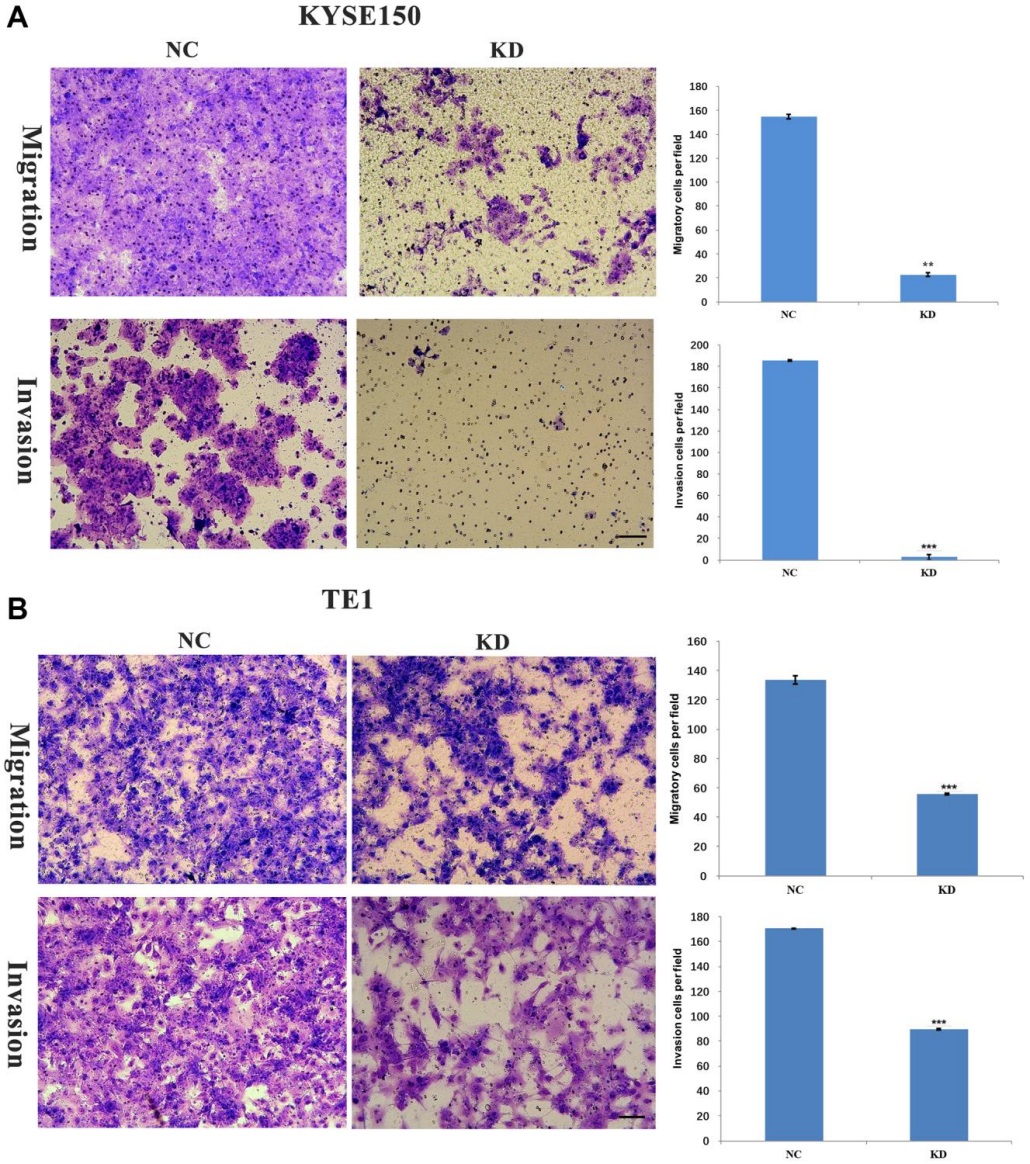
**KIF4A facilitated invasion and migration of ESCC cells.** Transwell assay was performed to compare migration and invasion between cells treated with shRNAs and NC. The representative images of migrated and invasive cells are shown (**A**–**B**) (scale bar 50 um). The presented figures are representative data from at least three independent experiments. ^*^P < 0.05, ^**^P < 0.01, and ^***^P < 0.001 for statistical analysis of the indicated groups.

An earlier study found that KIF4A expression was significantly associated with metastasis and prognosis in HCC patients [26]. Our data revealed that the expression of KIF4A was associated with distant metastasis in ESCC patients. By studying the cell movement of ESCC after KIF4A knockdown, the effect of KIF4A on the migration and invasion of ESCC cells was explored. The results showed that invading and migrating ESCC cells were significantly inhibited (Figure 6A–6B). These results indicated that KIF4A promoted migration and invasion of ESCC cells.

### KIF4A induced EMT and the MAPK and PI3K/AKT pathway in ESCC cells

**Figure 7:**
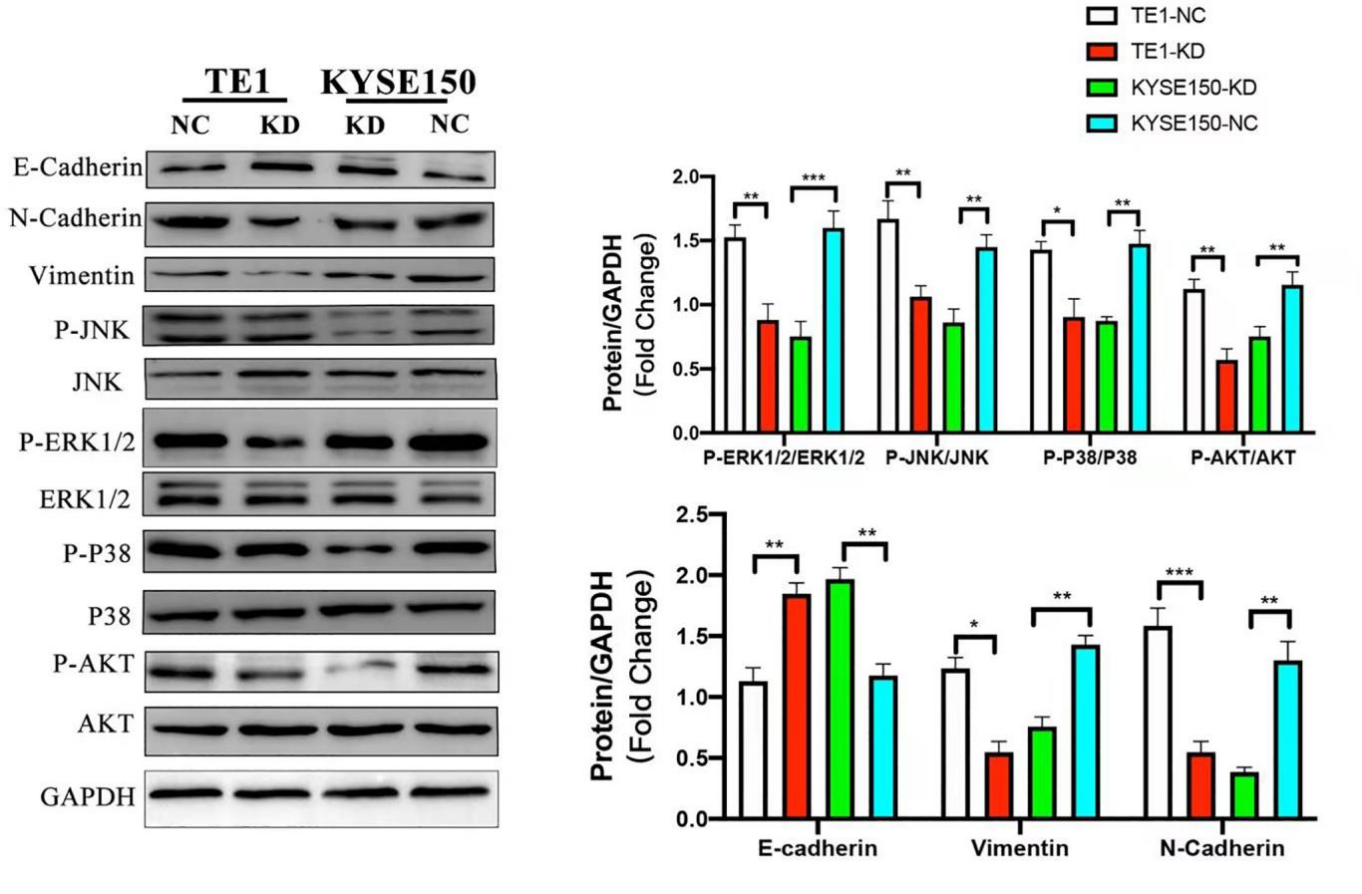
**KIF4A promoted PI3K/AKT and MAPK pathways and EMT in ESCC cells.** Comparing shRNA KIF4A with their respective control cells (NC) were seen in relative expression of P-AKT, AKT, P-ERK1/2, ERK1/2, P-JNK, JNK, P-P38, P38, and GAPDH was taken as control by WB. In addition, E-cadherin, N-cadherin, and vimentin were also detected by WB. Data represent mean ± SD. ^*^p < 0.05, ^**^p < 0.01, ^***^p < 0.001, *n* = 3.

We used WB to test the expression levels of phosphorylated AKT and P38, ERK1/2 and JNK after knockdown of exogenous KIF4A in ESCC cells, with the purpose of studying the effect of KIF4A on PI3K/AKT and MAPK pathways, and the results showed that all the markers above decreased in KIF4A knockdown cells. EMT plays a crucial role in migration and invasion of tumor cells derived from epithelial cells. We detected EMT hallmark using WB, and we discovered increased expression of E-cadherin (E-cad) and decreased expression of vimentin and N-cadherin (N-cad) after exogenous KIF4A knockdown in ESCC cells. The results were all shown in Figure 7. Overall, our findings showed that KIF4A promoted tumorigenesis and metastasis through the promotion of the PI3K/AKT and MAPK pathway and EMT in ESCC, respectively.

### KIF4A promoted ESCC tumor xenograft growth *in vivo*

**Figure 8:**
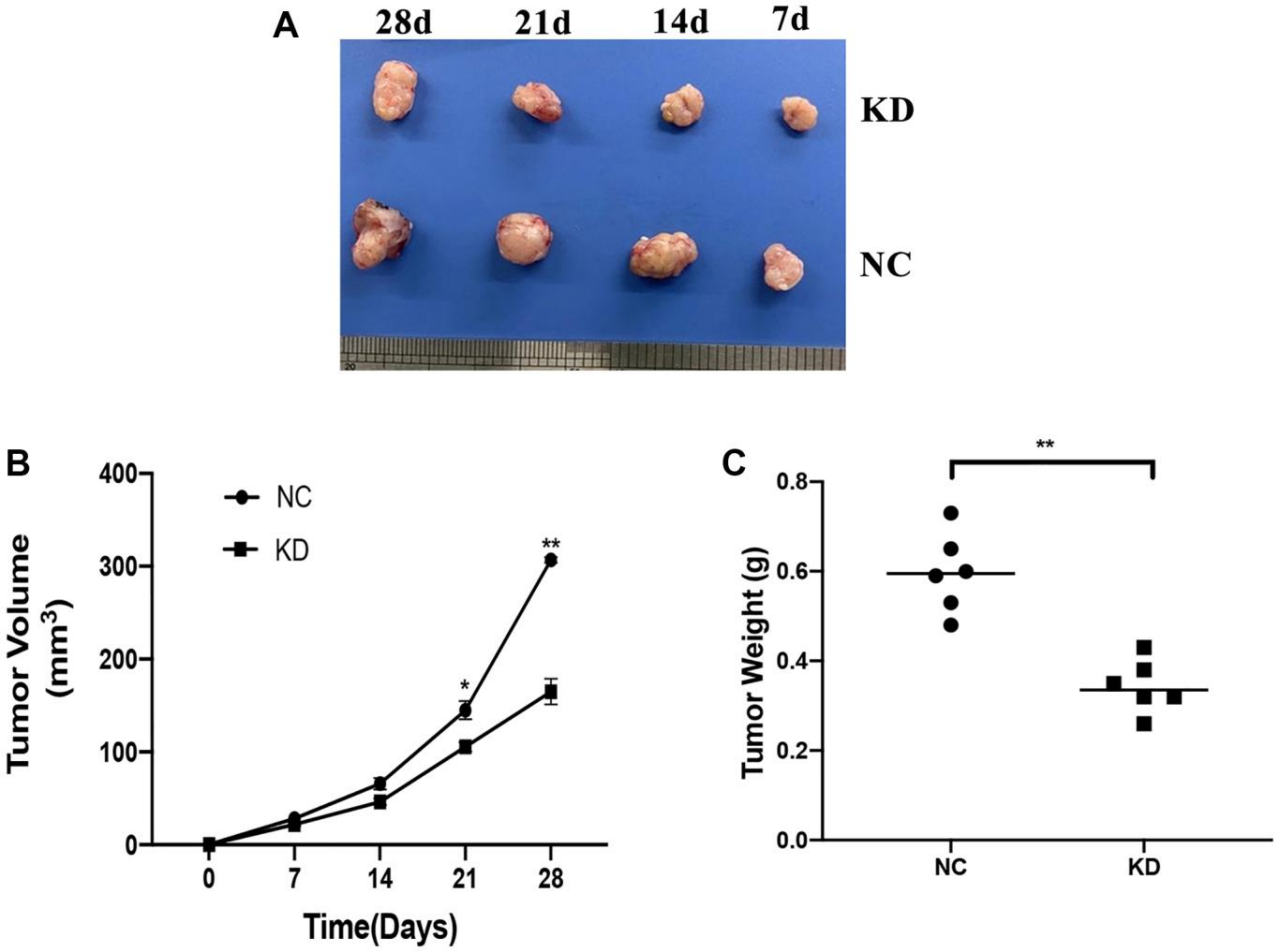
**Effects of KIF4A on ESCC cell growth *in vivo*.** Image of xenograft tumors (**A**). Growth curves of xenograft tumors formed by indicated KYSE150 cells in nude mice (**B**). Weight of xenograft tumors (**C**). Data represent mean ± SD (*n* = 6). ^*^p < 0.05, ^**^p < 0.01, ^***^p < 0.001.

The role of KIF4A in the development of the ESCC *in vivo* was assessed in a xenograft mouse model. The KYSE150 cells which stably expressing shCtrl or shKIF4A were injected subcutaneously into nude mice at the same dose. As illustrated by Figure 8, knockdown KIF4A suppressed the tumor xenografts growth in mice and reduced the tumor volumes and weights (Figure 8A–8C). The findings demonstrated that KIF4A knockdown suppressed tumor growth of ESCC *in vivo*.

## DISCUSSION

Esophageal squamous cell carcinoma (ESCC) is a global common cancer, particularly in some parts of Asia [1, 2]. The exact molecular mechanisms behind ESCC are still incompletely defined, leading to a poor ESCC prognosis. Resolving these concerns can be accomplished by analyzing three GEO mRNA microarray datasets to identify DEGs between primary tumor tissues (ESCC) and matched adjacent normal esophageal mucosa. Functional enrichment analyses were performed by GO and KEGG analyses. Then, we constructed a PPI network to visualize interactions of DEGs. Eventually, pathological staging and prognostic analysis of hub gene expression were carried out. Ultimately, we uncovered that high KIF4A expression implied a poor ESCC prognosis and revealed that KIF4A facilitated proliferation, migration, and invasion of ESCC *in vivo* and *in vitro*. Overall, these results indicated that KIF4A had a significant function as an oncogene in ESCC progression.

KIF4A, the vital subfamily of kinesin superfamily proteins (KIFs), is essential to regulate mitosis and meiosis, spindle organization and cytokinesis, and promotes proper axon growth in neuronal cells [9–11]. Interestingly, KIF4A is overexpressed in various cancers. Nevertheless, it is downregulated in gastric cancer by inhibiting gastric cancer cell proliferation [17], suggesting its distinct functions and mechanisms for different tumors. Previous studies have demonstrated that high KIF4A expression levels are associated with poor patient prognosis [10, 18]. Similarly, our study showed that KIF4A was upregulated in ESCC tissues relative to normal matched tissues and ESCC cell lines. Moreover, our follow-up analysis exhibited that upregulation of KIF4A expression was positively linked to increased invasion depth, tumor diameter, lymph node metastasis and advanced TNM stage. Moreover, multivariate Cox regression further demonstrated that KIF4A could be an independent predictor of poor prognosis in ESCC patients.

ESCC progression is characterized by excessive proliferation, avoiding programmed cell death, and infinite invasion and metastasis of cancer cells [19]. Previous studies have reported that KIF4A is involved in proliferation, apoptosis, and metastasis of cancer cells [20, 21]. From our data, ESCC cell proliferation and colony formation were downregulated by KIF4A knockdown in cultured ESCC cells. Additionally, KIF4A knockdown significantly restricted tumor growth *in vivo*. KIF4A downregulation decreased CRC cell lines relative to regulate p21 to promote CRC cell cycle progression at G1/S transition and by overexpression of cyclin D1-Cdk2/Cdk4 and cyclin E-Cdk2 directly implicate cyclins in RCC cell lines [20, 22]. Given our findings, Down-regulation of KIF4A significantly increases the percentage of cells in G0/G1 phase, while the distribution of cells in S phase and G2 phase decreases. Unfortunately, we did not detect the expression of p21, p27, Cdk2, Cdk4, and Cdk6 proteins. In addition, decreasing apoptosis is also a hallmark of cancer progression [23]. Yet, there was no evidence that KIF4A was involved in regulating ESCC cells’ apoptosis in our study.

To profoundly study the underlying mechanisms of ESCC, GO and KEGG pathway enrichment analyses were conducted to identify functional processes and pathways. Furthermore, PI3K pathway holds a significant function in cell cycle entry and glucose metabolism [24]. AKT phosphorylation promotes tumorigenesis via several oncogenic events, including apoptosis and cell proliferation [25]. KIF4A enhanced cell proliferation via activating Akt signaling in hepatocellular carcinoma; meanwhile [26], KIF4A downregulation dramatically decreased the expression of p-AKT in colorectal cancer [27]. Our data showed that KIF4A knockdown obviously decreased p-AKT expression in ESCC cell lines. MAPK had a dozen signaling pathways involved in cell growth, differentiation, proliferation, and apoptosis [28]. However, three major signaling pathways have been well-studied until now: Extra cellular regulated protein kinases (ERK) transduction pathway, P38 mitogen~ activated protein kinase MAPK pathway, and C**-**Jun-terminal kinase (JNK) transduction pathway. In particular, ERK signaling pathway is often upregulated in many tumors, mainly cell proliferation [29]. P38 transduction pathway is the main pathway involved in apoptosis initiation. After activated by extracellular stimulation, it exerts its biological effect and participates in differentiation, proliferation and apoptosis of cells [30]. JNK signaling pathway activation regulates a series of intracellular responses including cell differentiation, apoptosis and stress response [31]. Meanwhile, our study demonstrated similar results.

EMT is a feature of epithelial cells transforming into mesenchymal phenotype cells through specific procedures, holding a crucial role in migration and invasion of tumor cells derived from epithelial cells [32, 33]. An important hallmark of EMT is decreased expression of E-cadherin (E-cad) and increased expression of vimentin. In our study, transwell experiments revealed that ESCC cell migration and invasion were inhibited by KIF4A silencing. Moreover, E-cadherin level is downregulated, and vimentin expression is up after KIF4A knockdown, suggesting that KIF4A could promote tumor invasion and metastasis through EMT *in vitro*.

To this end, our study showed that KIF4A was upregulated in ESCC and significantly correlated with a poorer prognosis for the first time. KIF4A facilitated proliferation by PI3K/AKT, and MAPK signaling pathways and promoted migration and invasion through EMT. These findings shed light on KIF4A prospects as a prognostic ESCC biomarker. However, how KIF4A is activated and how it affects downstream pathways to achieve the function remains unclear.

## MATERIALS AND METHODS

### Human ESCC samples

The human research was approved by the principles of the Declaration of Helsinki and the local ethics committee of East Hospital Affiliated to Tongji University. Samples of Primary ESCC tumor and paired adjacent normal tissue were collected from 65 patients who had an oesophagectomy at the Department of Thoracic Surgery in East Hospital (Shanghai, China) from March 2014 to March 2016. None of the patients had a history of radiotherapy or chemotherapy before surgery. In accordance with WHO standards, each specimen underwent histological review and evaluation by two senior pathologists.

### Data collection

In the present study, three gene expression profile datasets (GSE17351 [35], GSE20347 [36], and GSE100942 [37]) of ESCC were obtained from Gene Expression Omnibus (GEO, https://www.ncbi.nlm.nih.gov/geo) database [34]. GSE17351, GSE20347, and GSE100942 contain 5, 17, and 4 pairs of tissues, respectively, including primary tumor tissues (ESCC) and paired nontumor tissues (normal tissues or adjacent normal esophageal mucosa).

### Identification of DEGs

We removed the probe sets that cannot match any of the gene symbols and averaged the genes that correspond to more than one probe set and if the dataset needs to be normalized, then normalized it using the normalize Between Arrays function in the limma R package. The sva R package was employed to remove batch effects across different platforms. The three datasets were then integrated into a whole dataset for analyzing the following DEGs. The limma R package analyzed DEGs between paired samples by paired test. The DEGs were defined as |logFC| ≥2 and adj. *P* < 0.001.

### KEGG and GO enrichment analyses of DEGs

Regarding common DEGs function, significant gene ontology and Kyoto Encyclopedia of Genes and Genomes enrichment analysis (*P* < 0.05) were performed by R package clusterProfiler [38, 39].

### PPI network construction and cytoHubba module analysis

In this study, PPI network of DEGs was constructed using STRING database (https://string-db.org/) [40], and a combined score of interaction >0.4 was considered statistically meaningful [41]. We then used cytoHubba module of Cytoscape (version 3.4.0) to screen key genes, predicting and exploring significant nodes and subnet of a given network through a number of topological algorithms. We applied the three most common topological algorithms (Maximal Clique Centrality, Density of Maximum Neighborhood Component and Degree) to filter the top 20 genes. Finally, we took the intersection of 60 genes and got six hub genes.

### Pathological staging and prognostic analysis of hub gene expression

For prognostic analysis, GEPIA2 and UALCAN analysis were used to analyze the overall survival, and *P*-value <0.05 was considered to be significantly related to prognosis. Besides, UALCAN analysis was utilized to analyze the relationship between KIF4A and N categories and clinical staging.

### Analysis of the role of prognostic hub genes in EC invasion

A further dataset, GSE21293 [42], which comprising gene expression data for invasive and non-invasive ESCC cells, was evaluated based on the GEO database to explore the role of hub DEGs in EC invasion.

### Establishment of KIF4A-knockdown ESCC cells

Lentiviral which comprising shRNAs targeting KIF4A was provided by GeneChem (GeneChem Co., Ltd., Shanghai, China) and transfected into KYSE150 and TE1 cells as directed by the manufacturer. Stable cell lines were screened with puromycin as described above. KIF4A shRNA targets were CCCTTTACAGAACA GACTA. The cells which transfected with scrambled shRNA were taken as NC.

### Reverse-transcription quantitative PCR (qPCR)

Total RNA was extracted from ESCC patients and their paired adjacent normal tissues using Trizol (Takara). Total RNA (1 μg) was reversely transcribed into cDNA with PrimeScriptTM RT Reagent Kit (Takara). qPCR was carried out using SYBR Green (Takara), and the results were normalized to GAPDH expression. The primer sequence of KIF4A is FORWARD (5′-3′) TGCCAACAAGCGTCTCAAGGATG, REVERSE (5′-3′) TCCATTCCACGGCTCTGAGTCTC, and that of GAPDH is FORWARD (5′-3′) AATGGGCAGCCGTTAGGAAA, REVERSE (5′-3′) GCGCCCAATACGACCAAATC.

### Western blot analysis

Proteins of esophageal tissue of ESCC patients, paired adjacent normal tissues, and cultured cells were extracted with RIPA lysis buffer (Thermo Fisher Scientific Inc), supplemented with Protease and Phosphatase Inhibitor Cocktail (Thermo Fisher Scientific Inc) and were quantified utilizing BCA Protein Assay Kit (Thermo Scientific). Antibodies of KIF4A (Abcam, ab122227), AKT (CST, 4685S), P-AKT (CST, 4060S), P38 (CST, 8690S), P-P38 (CST, 4511S), ERK1/2 (Abcam, ab17942), P -ERK1/2 (Abcam, ab214362), JNK (Abcam, Ab179461), P-JNK (Abcam, Ab124956), GAPDH (Abcam, ab8245) were incubated overnight at 4 degrees overnight, and then with horseradish peroxidase-conjugated secondary antibodies (Santa Cruz Biotechnology ) for 1 hr at room temperature.

### Immunohistochemical staining

Esophageal tissue of ESCC patients and paired adjacent normal tissues were fixed with 4% paraformaldehyde for 24 hours, and then the fixed tissues were paraffin-embedded and sliced into four μM sections. Immunohistochemical staining was performed with an antibody KIF4A (Abcam, ab122227). Two pathologists evaluated all slides blindly and independently. Tumor and normal tissues were categorized as KIF4A overexpression and low expression according to whether cancer cells with KIF4A staining was ≥5%.

### Cell culture

Human ESCC cell lines (KYSE150, KYSE410, KYSE140, and KYSE180) were purchased from Chinese Academy of Sciences Cell Cultures Library (Shanghai, China), while ESCC cell lines TE-1 was provided by American Type Culture Collection (Manassas, VA, USA). The human immortalized esophageal epithelial cell line NE1 was graciously provided by Professor Yongxin Zhou (Shanghai, China). All cell lines were cultured in RPMI 1640 medium at 37°C in 5% CO2 and 50% humidity.

### Cell proliferation assay

TE-1 and KYSE150 were cultured in 96-well plates at 5 × 10^3^ in serum starvation overnight. Each well was handled with 10 μL reagents from Cell Counting Kit-8 (Beyotime Biotechnology, Shanghai, China) and then each well was supplemented with 10 μL CCK-8 and incubated at 37°C for 2 h. Three auxiliary holes in each group. Optical density (OD) was measured at 450 nm using a microplate reader (BioTek, USA) [43]. The cells were transfected by shRNA for 48 hours and then counted.

### Flow cytometry for cell cycle and apoptosis analysis

After 48 hours of transfection, synchronize the cells through serum starvation overnight and induce re-entry into the cell cycle by incubating for 4 hours. Fix the collected cells in precooled 70% ethanol at 4°C overnight, and then resuspend them with RNaseA at 37°C for 30 minutes. Subsequently stained with propidium iodide (PI) in a dark environment at 4°C for 30 minutes. Apoptosis assay was carried out using Annexin V-PE apoptosis detection kit (Beyotime Biotechnology, Shanghai, China) as per manufacturer’s protocol. After 48 hours of transfection with shRNA, the cells were stained with membrane Annexin V and PE for 15 minutes, and then analyzed by flow cytometry (Beckman Coulter, USA).

### Transwell migration and invasion assays

Briefly, the upper chamber is filled with 1 × 10^5^ cells, 200 μL of fetal bovine serum (FBS)-free medium is added, and the lower chamber with 600 μL of 80% FBS-containing medium. Then the cell line was fixed with 10% formalin for 15 minutes and stained with 0.25% crystal violet for 15 minutes, rinsed again with sterile water. Finally Counting the migrating cells in 5 photographed areas at 200 × magnification.

### Colony formation assay

For colony formation analysis, we inoculated 200 cells on each 6-well plate. After 14 days of culture in RPMI1640 medium with 10% FBS, crystal violet staining was used to count cell colonies. We repeated experiments at least three times, and data were represented as mean ± SEM.

### Mouse xenograft tumor model

BALB/c nude mice (aged 6–8 weeks) were purchased from Beijing Vital River Laboratory Animal Technology Corporation. Mice experiments were approved by Tongji Hospital Affiliated to Tongji University, Animal Care Committee of Tongji Hospital Affiliated to Tongji University. Stable KIF4A knockdown cells and control Kyse150 cells (5 × 10^6^) were injected into the mice’s armpits. The volume (V) of formed tumor was computed using the formula V = L × W^2^/2 (L: long axis; M: short axis). Twenty-one days after the injection, the nude mice were sacrificed, and the tumors were dissected and weighed.

### Data analysis

The results are represented as mean ± SEM. Two-tailed Student’s tests were used for two-group comparisons, and ANOVA followed by post hoc Tukey’s test was used for multiple-group comparisons. GraphPad Prism 8.0 (San Diego, CA, USA) and SPSS 22.0 (New York, USA) statistical software are used for data analysis. A *p*-value less than 0.05 was considered statistically significant.

## Supplementary Materials

Supplementary Figures
