# Supplementary material for: Identification of KIF4A as a prognostic biomarker for esophageal squamous cell carcinoma
Source: Aging (Albany NY). 2021 Nov 14;13(21):24050–70. doi: 10.18632/aging.203585 (PMC8610135; doi:10.18632/aging.203585)
Supplement: Supplementary Figures [file aging-13-203585-s001.pdf]

Supplementary Figures

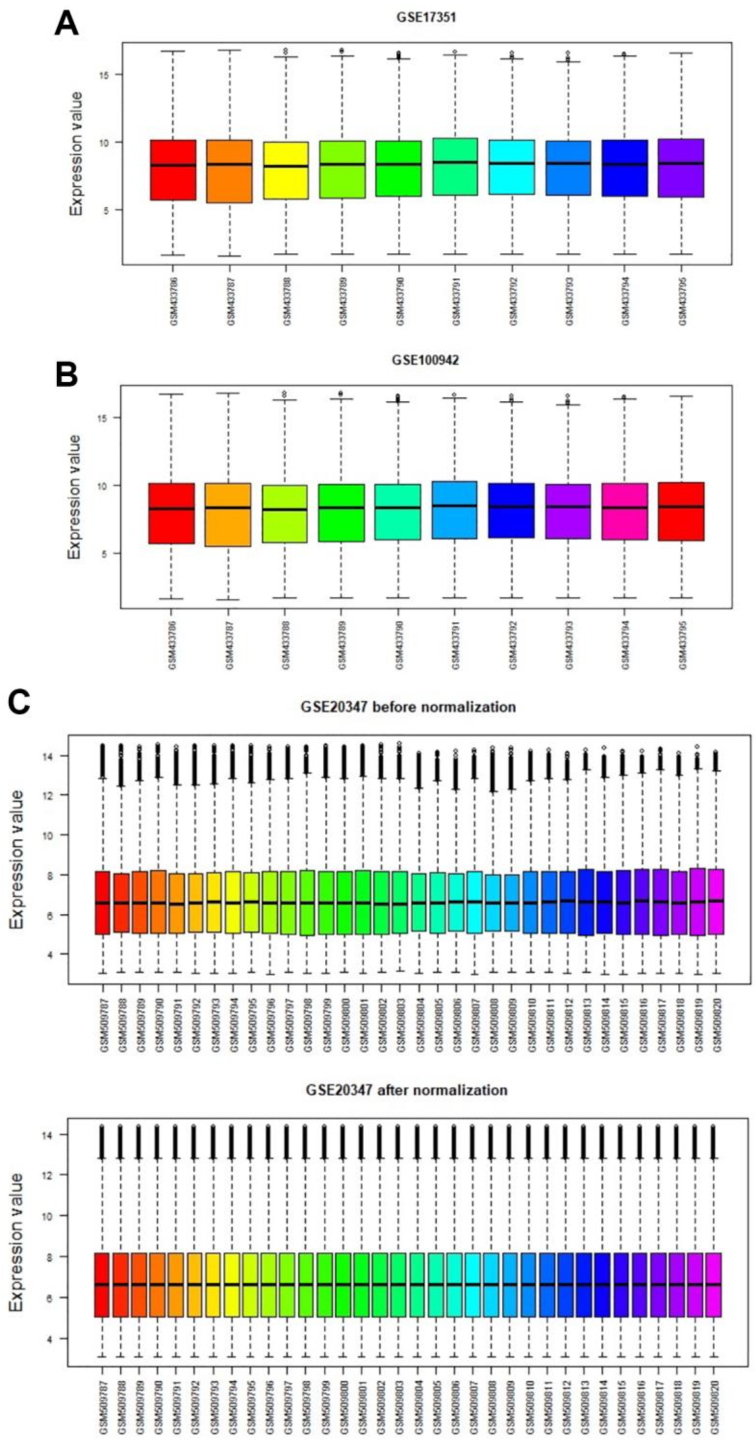

Supplementary Figure 1. Normalization of the three datasets. Including GSE17351 (**A**), GSE100942 (**B**), GSE20347 (**C**).

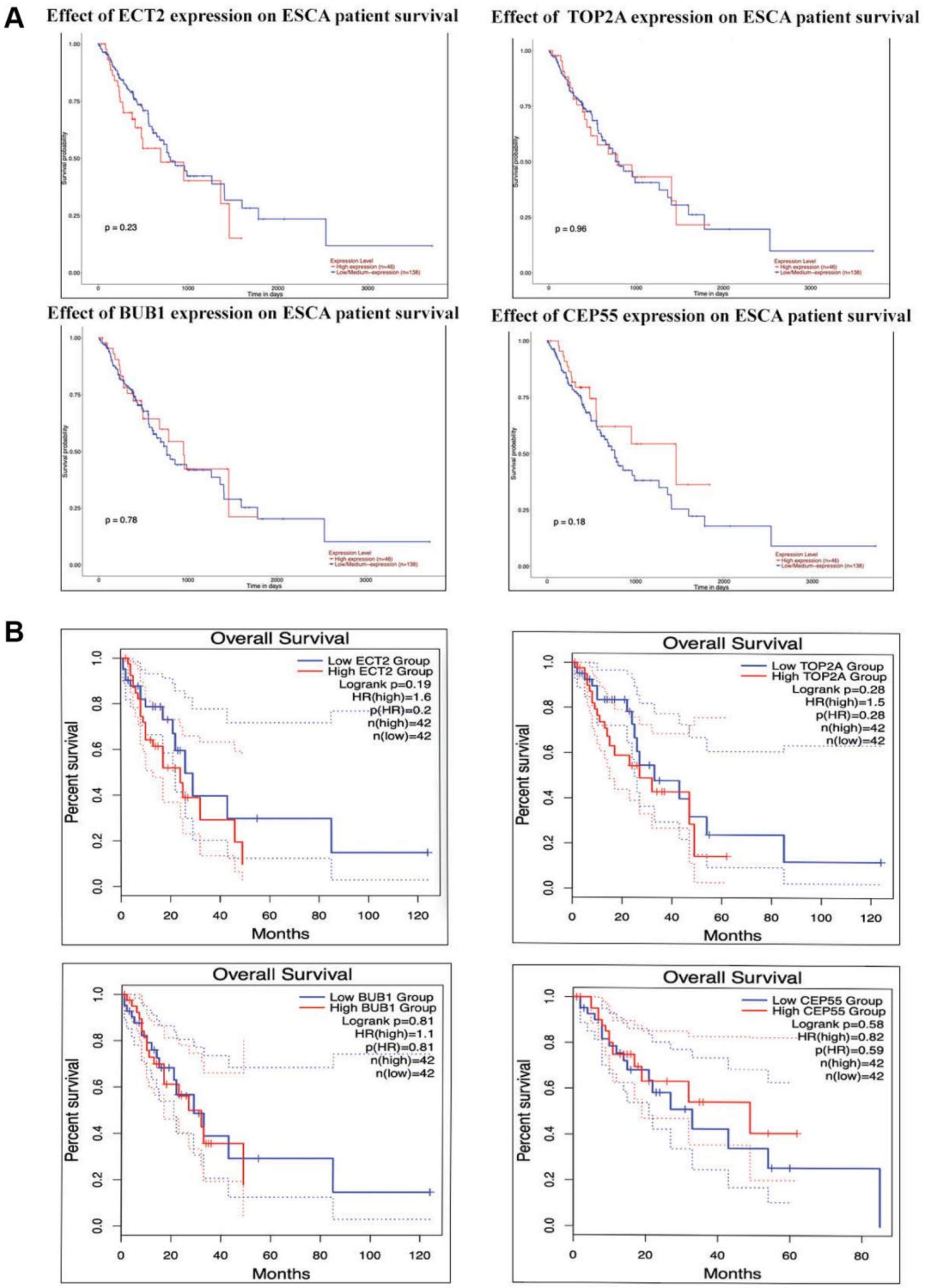

Supplementary Figure 2. Effects of ECT2, TOP2A, BUB1 and CEP55 expression on OS. The relationship between ECT2, TOP2A, BUB1 and CEP55 expression and OS for patients with ESCC by UALCAN (**A**) and GEPIA2 (**B**) analysis.

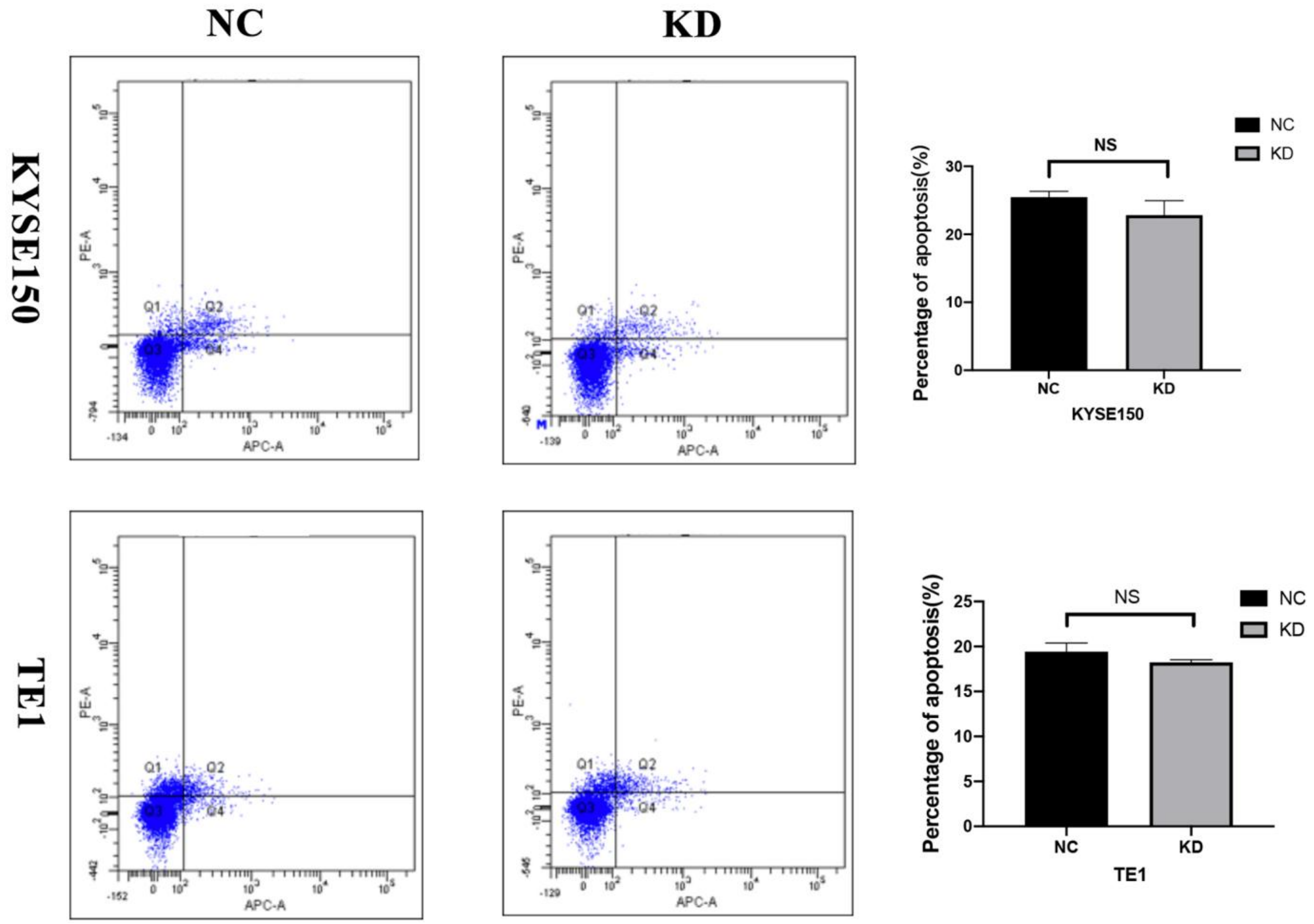

Supplementary Figure 3. Effects of KIF4A on ESCC cell apoptosis. The Representative images of Annexin V-PE double staining of KYSE-150 and TE-1 cells transfected with control or KIF4A shRNAs for 48 h were determined by flow cytometry. The data are presented as the means ± SD. NS: No statistically significant difference.
